# Hypoxia Reduces the Efficiency of Elisidepsin by Inhibiting Hydroxylation and Altering the Structure of Lipid Rafts

**DOI:** 10.3390/md11124858

**Published:** 2013-12-02

**Authors:** Anna Király, Tímea Váradi, Tímea Hajdu, Ralph Rühl, Carlos M. Galmarini, János Szöllősi, Peter Nagy

**Affiliations:** 1 Department of Biophysics and Cell Biology, University of Debrecen, Nagyerdei krt. 98, Debrecen 4032, Hungary; E-Mails: anna.kiraly.aok@gmail.com (A.K.); tvaradi@med.unideb.hu (T.V.); h.timea@hotmail.com (T.H.); szollo@med.unideb.hu (J.S.); 2 Department of Biochemistry and Molecular Biology, University of Debrecen, Nagyerdei krt. 98, Debrecen 4032, Hungary; E-Mail: ralphruehl@web.de; 3 Cell Biology Department, PharmaMar, Avda de los Reyes 1, Pol. Ind. La Mina, Colmenar Viejo, Madrid 28770, Spain; E-Mail: cgalmarini@pharmamar.com; 4 MTA-DE Cell Biology and Signaling Research Group, University of Debrecen, Nagyerdei krt. 98, Debrecen 4032, Hungary

**Keywords:** elisidepsin, lipid rafts, hydroxylated lipids, fatty acid 2-hydroxylase, cooperative binding, membrane permeabilization

## Abstract

The mechanism of action of elisidepsin (PM02734, Irvalec^®^) is assumed to involve membrane permeabilization via attacking lipid rafts and hydroxylated lipids. Here we investigate the role of hypoxia in the mechanism of action of elisidepsin. Culturing under hypoxic conditions increased the half-maximal inhibitory concentration and decreased the drug’s binding to almost all cell lines which was reversed by incubation of cells with 2-hydroxy palmitic acid. The expression of fatty acid 2-hydroxylase was strongly correlated with the efficiency of the drug and inversely correlated with the effect of hypoxia. Number and brightness analysis and fluorescence anisotropy experiments showed that hypoxia decreased the clustering of lipid rafts and altered the structure of the plasma membrane. Although the binding of elisidepsin to the membrane is non-cooperative, its membrane permeabilizing effect is characterized by a Hill coefficient of ~3.3. The latter finding is in agreement with elisidepsin-induced clusters of lipid raft-anchored GFP visualized by confocal microscopy. We propose that the concentration of elisidepsin needs to reach a critical level in the membrane above which elisidepsin induces the disruption of the cell membrane. Testing for tumor hypoxia or the density of hydroxylated lipids could be an interesting strategy to increase the efficiency of elisidepsin.

## 1. Introduction

Although significant progress has been made in the understanding of cancer at the molecular and cellular level, the potency of chemotherapy of advanced malignant tumors is still limited and based on conventional cytotoxic drugs calling for medications with new mechanism of action [1]. Elisidepsin (Irvalec^®^, PM02734) is a synthetic cyclodepsipeptide closely related to Kahalalide F, a natural antitumor compound isolated from the Hawaiian marine mollusk Elysia rufescens [2,3,4]. Preclinically, elisidepsin showed antiproliferative activity against a broad spectrum of tumor types [5]. Additionally, elisidepsin has been found to have synergistic effects when combined with several different conventional chemotherapeutic agents and tyrosine kinase inhibitors in cell lines and mouse xenograft models most likely due to its unique mechanism of action [6,7]. In clinical trials, elisidepsin has been shown to have a low toxicity profile [8,9,10,11] and preliminary assessment of its clinical efficacy showed interesting results in different solid tumors [8,9,10,11,12]. 

Although ErbB proteins have been implicated as the target of elisidepsin based on weak correlations between the drug’s efficiency and ErbB protein expression levels [6,7,13], we have refuted this hypothesis by showing that the expression of ErbB1, ErbB2 or ErbB3 proteins have no influence on the sensitivity of cell lines to elisidepsin [14]. According to the most widely accepted view the primary mechanism of action of elisidepsin involves a direct hit on the membrane by binding to lipid rafts [5,14]. Based on experiments with RNA interference-mediated knock-down of fatty acid 2-hydroxylase (FA2H) and incorporation of exogenous hydroxylated fatty acids, 2-hydroxylated sphingolipids have been suggested as the binding site of elisidepsin [15]. It has been postulated that elisidepsin binds to hydroxylated lipids in rafts and induces rapid permeabilization of the cell membrane [14]. All other effects of the drug, including autophagy [16], necrosis [2,13], disruption of lysosomal membranes [17], inhibition of Akt signaling [6,16] and downregulation of ErbB3 expression and activation [13,14], are thought to be secondary effects pursuant to the primary hit on the membrane.

As FA2H, the enzyme thought to be responsible for the generation of the target of elisidepsin [15], is oxygen dependent [18,19] and tumor hypoxia is widespread in advanced tumors [20], we have decided to investigate its role in the mechanism of action of elisidepsin. In the current paper, we show that the sensitivity of cell lines to elisidepsin is proportional to their FA2H level and hypoxia reduces the efficiency of the drug. The effect of hypoxia was reversed by the addition of exogenous hydroxylated fatty acid. Hypoxia was found to induce changes in the organization of lipid rafts. Our results suggest that testing for tumor hypoxia or the density of hydroxylated lipids could potentially increase the efficacy of elisidepsin.

## 2. Results

### 2.1. The Elisidepsin Sensitivity of Cell Lines is Reduced under Hypoxic Conditions

We suspected that hypoxia would lead to a diminished synthesis of hydroxylated fatty acids thereby reducing the sensitivity of cells to elisidepsin. In order to test the aforementioned assumption different cell types were cultured under hypoxic conditions for four days followed by testing their elisidepsin sensitivity. Four of the seven investigated cell lines (A431, CHO, HaCaT, HeLa) displayed significantly reduced elisidepsin sensitivity while the other three cell types (MCF-7, MDA-MB-453, SKBR-3) were resistant to the hypoxia-induced effects (Table 1, Supplementary Figure S1). We reasoned that the applied four-day hypoxia may be insufficient to deplete the pool of hydroxylated fatty acids in those cells which did not show any change in elisidepsin sensitivity in conditions of reduced oxygen partial pressure. Therefore, these cell types were cultured under hypoxic conditions for fourteen days followed by testing their elisidepsin sensitivity. Extensive hypoxia slightly increased the IC_50_ values in two of the cell lines (MDA-MB-453, SKBR-3), whereas MCF-7 cells did not show any change under these experimental conditions either (Table 1, Supplementary Figure S2). We conclude that hypoxic conditions generally lead to diminished sensitivity of cells to elisidepsin. 

**Table 1:** Sensitivity of cell lines to elisidepsin under hypoxic and normoxic conditions. Cells plated in 96-well plates were kept under hypoxic conditions for four days or fourteen days followed by elisidepsin treatment. The normoxic control samples were plated one day before adding elisidepsin. Both normoxic and hypoxic cells were treated with the drug for 30 min and allowed to grow for another three days under normoxic and hypoxic conditions. The mean IC_50_ values (±standard error of the mean), determined from three independent measurements, are shown in the table. Two-way ANOVA indicated a significant effect of hypoxia. Pairwise comparisons between normoxic and hypoxic samples were carried out by Tukey’s HSD test. * Asterisks indicate significant difference compared to the normoxic values (*p <* 0.05). Representative dose-response curves from which the IC_50_ values were determined are shown in Supplementary Figures S1 and S2 (n. d. = not determined).

Cell lines	IC_50_ (µM)		
	Normoxia	4-day hypoxia	14-day hypoxia
A431	7.8 ± 0.8	15.4 ± 1.8 *	n. d.
CHO	15 ± 0.8	66 ± 9.3 *	n. d.
HaCaT	7.5 ± 0.9	27.6 ± 4.2 *	n. d.
HeLa	9.1 ± 1.2	16.5 ± 1.7 *	n. d.
MCF-7	1.4 ± 0.3	1.6 ± 0.5	1.7 ± 0.6
MDA-MB-453	3.6 ± 0.5	3.9 ± 0.7	6.1 ± 0.5 *
SKBR-3	2.4 ± 0.4	2.3 ± 0.5	5.2 ± 0.2 *

### 2.2. The Expression of FA2H Correlates with Elisidepsin Sensitivity in Normoxia and Determines the Hypoxia-Induced Increase in the IC_50_ Values

**Figure 1:**
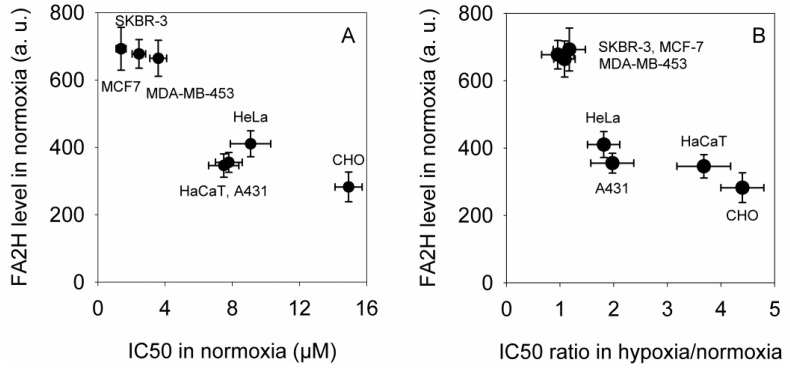
FA2H expression determines the sensitivity of cell lines to elisidepsin under normoxic conditions and the hypoxia-induced decrease in elisidepsin responsiveness. (**A**) The IC_50_ values shown in Table 1 were correlated with the expression level of FA2H determined by flow cytometry. The means (±standard error of the mean) of three independent measurements are shown; (**B**) The sensitivity of cell lines was determined under normoxic and hypoxic conditions as shown in Table 1. The FA2H expression level is plotted as a function of the ratio of the IC_50_ values determined under hypoxic and normoxic conditions. The means (±standard error of the mean) of three independent measurements are shown.

We assumed that the complete absence of hypoxia-induced changes in MCF-7 may be the consequence of the high expression level of FA2H in these cells. Therefore, we measured the expression level of FA2H by fluorescent staining followed by flow cytometry and correlated the fluorescence intensities with the IC_50_ values measured in normoxic conditions (Figure 1A). The IC_50_ values and the expression level of FA2H showed strong negative correlation (Pearson correlation coefficient: −0.9, 95% confidence interval: [−0.96, −0.78], Spearman rank correlation coefficient: −0.86, 95% confidence interval: [−0.94, −0.68]; confidence intervals determined by Fisher’s *z*-transform). These results confirmed previous data implying the role of FA2H in determining elisidepsin sensitivity [15]. Next, we compared the magnitude of the hypoxia-induced changes to the expression of FA2H (Figure 1B). The analysis revealed a strong correlation between the parameters (Pearson correlation coefficient: −0.85, 95% confidence interval: [−0.94, −0.66], Spearman rank correlation coefficient: −0.89, 95% confidence interval: [−0.96, −0.75], Figure 1B) indicating that high levels of FA2H (indicative of a higher density of hydroxylated lipids and thus high cytotoxic activity of elisidepsin) counteracts the effect of hypoxia in reducing the sensitivity of cells to the drug.

In order to show that hypoxia indeed decreases the amount of the product of FA2H we quantified 2-hydroxy fatty acids in normoxic and hypoxic cells using mass spectrometry. The results showed that culturing cells for four days under hypoxic conditions significantly decreased the amount of 2-hydroxylated palmitic and stearic acids without significantly affecting their 3-hydroxylated counterparts. Representative mass spectrometry tracks are shown in Supplementary Figure S3.

### 2.3. 2-Hydroxy Palmitic Acid Reverses the Effect of Hypoxia on Elisidepsin Sensitivity

Hypoxia may have resulted in a reduced concentration of hydroxylated fatty acids in the plasma membrane thereby bringing about the observed reduced efficiency of elisidepsin. In order to test the aforementioned assumption hypoxia-responsive cell lines (A431, CHO, HaCaT, HeLa) were incubated in the presence of 2-hydroxy palmitic acid during the last 24 hours of their hypoxic culture followed by testing their elisidepsin sensitivity. Hypoxia reduced the efficiency of elisidepsin compared to normoxia in this series of experiments as well, while 2-hydroxy palmitic acid reversed the hypoxia-induced changes (Table 2, Supplementary Figure S4). We also tested the effect of 3-hydroxy palmitic acid on one of the cell lines (A431), but it could not restore the elisidepsin sensitivity of hypoxic cells (data not shown). This finding is in accordance with the lack of any significant hypoxia-induced change in the levels of 3-hydroxy fatty acids (Supplementary Figure S3). These results imply that 3-hydroxy fatty acids do not play an important role in determining elisidepsin sensitivity. The fact that methanol, the solvent of the hydroxylated fatty acid, was without any significant effect allowed us to conclude that 2-hydroxy palmitic acid abolishes the effect of hypoxia on the elisidepsin sensitivity of the cell lines tested.

**Table 2:** The effect of hydroxylated palmitic acid on the sensitivity of cell lines to elisidepsin in hypoxia. Cells plated in 96-well plates were kept under hypoxic conditions for four days. On the third day, they were treated with 100 µM 2-hydroxy palmitic acid (2-OH-PA) or its solvent, methanol (MeOH). The normoxic control samples were plated one day before adding elisidepsin. Both normoxic and hypoxic cells were treated with the drug for 30 min and allowed to grow for another three days under normoxic and hypoxic conditions. The mean IC_50_ values (±standard error of the mean), determined from three independent measurements, are shown in the table. Representative dose-response curves from which the IC_50_ values were determined are shown in Supplementary Figure S4.

Cell lines	IC_50_ (µM)			
	Normoxia	Hypoxia	MeOH in hypoxia	2-OH-PA in hypoxia
A431	9.0 ± 1.7	17.9 ± 2.8	15.6 ± 3.1	7.75 ± 2.0
CHO	15.6 ± 2.1	34.6 ± 4.7	38.9 ± 5.3	12.3 ± 2.4
HaCaT	7.6 ± 1.9	18.7 ± 2.5	16.4 ± 2.8	9.5 ± 1.7
HeLa	9.4 ± 1.0	21.0 ± 3.3	19.0 ± 2.4	10.1 ± 1.8

### 2.4. Hypoxia Reduces the Binding of Fluorescent Elisidepsin

**Figure 2:**
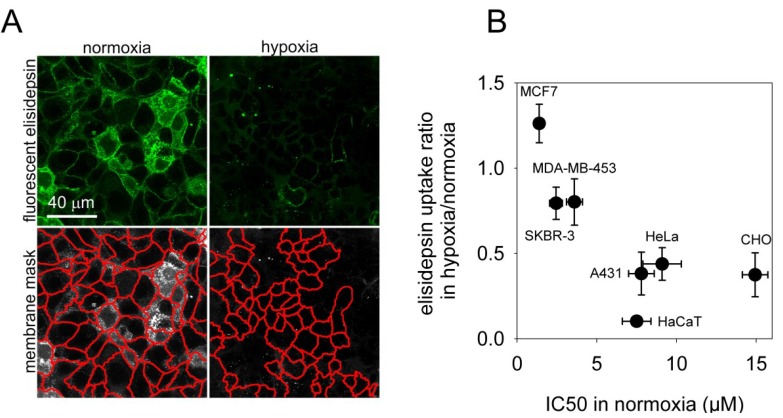
The binding of fluorescent elisidepsin is reduced by hypoxia. (**A**) A431 cells kept under hypoxic conditions for four days and their normoxic counterparts were labeled with a mixture of elisidepsin containing OregonGreen488-conjugated and unlabeled elisidepsin at a molar ratio of 1:4 for two min followed by washing and confocal microscopy in five min. The total concentration of elisidepsin was 2 µM, approximately 5-times smaller than the IC_50_ of A431 cells. The fluorescence intensity was evaluated in the membrane mask determined by manually-seeded watershed transformation after subtracting the background determined in a cell-free area of an image; (**B**) A mixture of OregonGreen488-elisidepsin and unlabeled elisidepsin (molar ratio of 1:4) was added to the cell suspension and the fluorescence intensity was immediately measured by flow cytometry.The total concentration of elisidepsin was 0.5 µM. The slope of cell-bound elisidepsin fluorescence intensity as a function of time was estimated in the first ~30 s of uptake and the relative reduction of the rate of elisidepsin binding in hypoxia is plotted against the IC_50_ of elisidepsin in normoxia (mean ± standard error of the mean, *n =* 3).

The results presented so far imply that hypoxia leads to diminished binding of elisidepsin to the cell membrane. In order to test this idea normoxic and hypoxic A431 cells were incubated in the presence of a 1:4 mixture of fluorescent and unlabeled elisidepsin for 2 min followed by removal of unbound elisidepsin and confocal microscopy. Dilution of fluorescent and non-fluorescent elisidepsin was necessary since it has been shown previously that fluorescent elisidepsin displays vague or no fluorescence in the membrane in the absence of unlabeled drug molecules most likely due to the formation of clusters and fluorescence quenching [5]. The fluorescence intensity was evaluated in the membrane after image segmentation showing a significant reduction in the binding of elisidepsin by hypoxia (mean fluorescence intensity in normoxic cells: 79 ± 7, in hypoxic cells: 27 ± 5; *p =* 0.0002; Figure 2A). Since generation of statistically reliable data is more straightforward in flow cytometry, we repeated the binding experiment using this technique. Elisidepsin binds to cells and is internalized very rapidly [14], therefore fluorescence intensities reported by the flow cytometer do not represent the amount of membrane-bound drug. In order to get around this problem we developed an approach to measure the kinetics of binding of fluorescent elisidepsin to the cells (see Supplementary Materials and Methods for details). According to this model, the very first part of the curve represents membrane-bound elisidepsin without significant contribution from the intracellular space. The slope of the initial part of the curve was shown to be proportional to the amount of membrane-bound elisidepsin. We compared the uptake of fluorescent elisidepsin in a panel of seven cell lines and calculated the fold-reduction induced by hypoxia, which was correlated with the IC_50_ values observed under normoxic conditions (Figure 2B). According to this analysis, hypoxia significantly reduced the binding of elisidepsin in those cell lines (A431, CHO, HaCaT, HeLa) whose IC_50_ values were increased under hypoxic conditions. The hypoxia-induced reduction in elisidepsin binding displayed a negative correlation with the normoxic IC_50_ values. We can conclude that the hypoxia-induced reduction in elisidepsin sensitivity is caused by reduced binding of the drug to the cell membrane under hypoxic conditions.

### 2.5. Elisidepsin Induces Clustering of GPI-Anchored GFP

**Figure 3:**
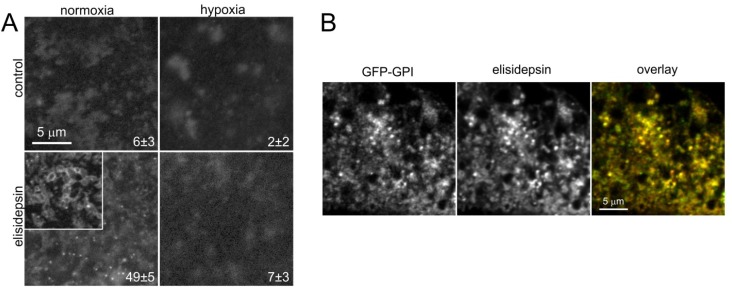
Elisidepsin induces the clustering of GPI-anchored GFP. (**A**) A431 cells were kept under hypoxic conditions for two days followed by transfection with GFP-GPI and another two days in hypoxia. Control normoxic cells were also transfected with GFP-GPI and kept under normoxic conditions for another two days. Confocal microscopic images were taken before and after treating the cells with 10 µM elisidepsin for five min. The representative images show the membrane adjacent to the coverslip. The insert in the lower left panel displays another normoxic cell treated with elisidepsin. Numbers in the lower right corner of images represent the mean (±standard error of the mean) number of bright fluorescent spots in an image determined from six images; (**B**) Normoxic A431 cells transfected with GFP-GPI were labeled with 2 µM elisidepsin containing AlexaFluor555-tagged and unlabeled elisidepsin at a molar ratio of 1:4 for two min followed by washing twice. Confocal microscopic images taken five min after the washing in the GFP and AlexaFluor555 channels and their overlay (green–GFP-GPI; red–elisidepsin) are shown in the figure.

All of the current results and evidence presented elsewhere [5,14,15] point at elisidepsin binding to the membrane, more specifically to lipid rafts. Therefore, we wanted to investigate the effect of elisidepsin on the distribution of lipid rafts in the membrane. To this aim, normoxic and hypoxic A431 cells were transfected with GPI-anchored GFP (GFP-GPI) followed by elisidepsin treatment in two days. The fluorescence of GFP-GPI was unevenly distributed in the membrane of both normoxic and hypoxic cells. Elisidepsin treatment induced the formation of bright fluorescent spots in normoxic cells while it was without any significant effect in hypoxic cells (Figure 3A). The number of bright fluorescent clusters, enumerated by an algorithm, was shown to be significantly higher in elisidepsin-treated normoxic cells than under other conditions (two-way ANOVA followed by Tukey’s HSD test, *p <* 0.01). Next, we incubated GFP-GPI-transfected cells in the presence of a fluorescent analog of elisidepsin for two min followed by determining the colocalization between the two fluorescent signals. Quantitative analysis revealed a strong correlation between the distribution of GFP-GPI and elisidepsin (Figure 3B, correlation coefficient = 0.92, 95% confidence interval = [0.78,0.97]).

**Figure 4:**
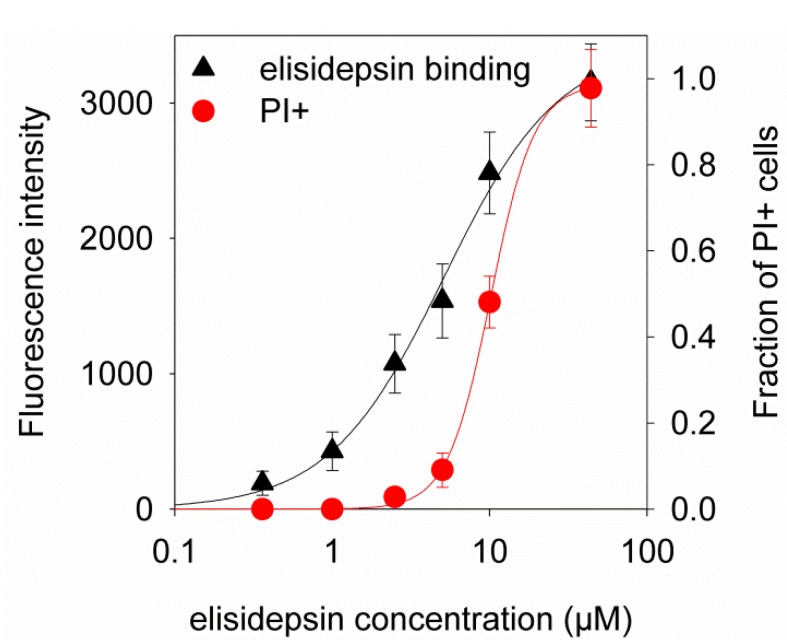
Difference in the concentration dependence of the binding and killing effect of elisidepsin. A431 cells were treated with six different concentrations of elisidepsin containing OregonGreen488-elisidepsin and unlabeled elisidepsin mixed at a molar ratio of 1:4 in the presence of 10 µg/mL propidium iodide. After a 20-min treatment, cells were washed and imaged using confocal microscopy. The background-corrected fluorescence intensity of membrane-bound elisidepsin (triangles) and the fraction of propidium iodide-positive cells (circles) were determined and plotted as a function of elisidepsin concentration. Error bars represent the standard error of the mean. The continuous lines are fits of the Hill equation to the measurement points. A representative image series is shown in Supplementary Figure S5.

Since we observed binding of fluorescent elisidepsin at concentrations that did not induce any killing, we systematically analyzed the reason for this discrepancy. As the IC_50_ of fluorescent elisidepsin was found to be identical to that of the unconjugated drug within experimental error (IC_50_ of unconjugated drug in A431 cells: 8.8 ± 1.6 µM, fluorescent analog: 9.2 ± 1.8 µM; *p >* 0.1), we compared the concentration dependence of killing and the binding of fluorescent elisidepsin. Killing was quantitated as the fraction of propidium iodide-positive cells and binding of fluorescent elisidepsin was determined in the membrane. The curves were fitted separately allowing for different half-maximal effective or inhibitory concentrations (*K*_d_ of binding and IC_50_ for killing) and Hill coefficients. The *K*_d_ of binding turned out to be 5.1 µM, whereas the IC_50_ value was found to be 10.2 µM in agreement with previous analyses. As opposed to the binding of fluorescent elisidepsin, which was non-cooperative characterized by a Hill coefficient of 1.1, the killing curve was fitted with an equation with a Hill coefficient of 3.2 (Figure 4, Supplementary Figure S5). These observations support the assumption that elisidepsin undergoes oligomerization in the membrane accompanied by increased clustering of lipid raft-associated proteins.

### 2.6. Hypoxia Decreases the Clustering of Lipid Rafts and Induces Changes in the Fluidity and Compactness of the Membrane

**Figure 5:**
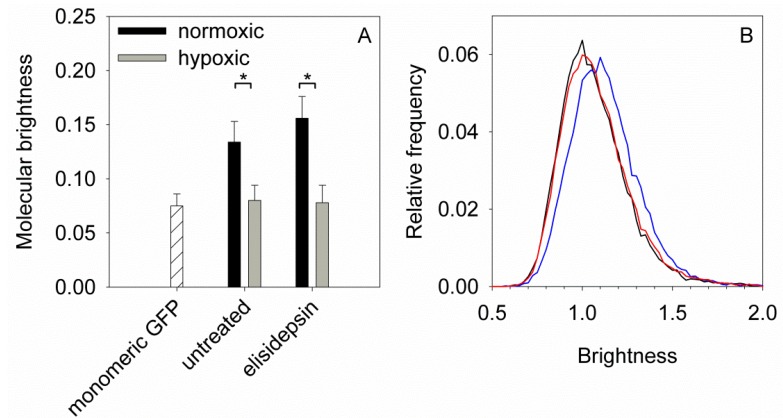
(**A**) A431 cells were cultured under normoxic conditions or kept in a hypoxic atmosphere for four days. Both normoxic and hypoxic cells were transfected with GPI-anchored GFP two days before N&B analysis using confocal microscopy. Cells were left untreated or incubated in the presence of 10 µg/mL elisidepsin for three min. The molecular brightness of GPI‑GFP (mean ± standard error of the mean), determined from ten cells, is shown in the graph.Asterisks indicate a significant difference between normoxic and hypoxic cells (*p <* 0.05, ANOVA followed by Tukey’s HSD test). The molecular brightness of monomeric soluble GFP is shown as a reference; (**B**) Representative brightness curves of pixels in normoxic (blue) and hypoxic (red) cells and in a sample containing soluble monomeric GFP (black).

Results presented in the previous sections imply that elisidepsin alters the distribution and clustering of lipid rafts. In order to test this hypothesis directly we carried out N&B analysis to determine the mean number of GPI-GFP molecules in a cluster. We did not find any significant difference between the molecular brightness of GPI-GFP in elisidepsin-treated and control cells implying that the drug did not significantly change the average number of GPI-GFP molecules per cluster (Figure 5). 

Hypoxia is expected to decrease the amount of hydroxylated lipids in the membrane thereby reducing the number of hydrogen bond donors and acceptors, which may cause measurable changes in the clustering of membrane proteins as well as in the fluidity and compactness of the membrane. According to N&B analysis carried out on GPI-GFP-transfected normoxic and hypoxic A431 cells the molecular brightness of GPI-GFP was indicative of molecular dimers in normoxic cells while a pure monomeric population was present in hypoxic cells (Figure 5). Since our previous results pointed at an effect of elisidepsin on the structure of the plasma membrane [14], we analyzed whether hypoxia modifies the fluidity and compactness of the membrane. Both the viscosity (measured by fluorescence anisotropy) and the order (measured by the generalized polarization of Laurdan) of the plasma membrane increased in A431 cells cultured under hypoxic conditions for four days, while there was no change in these parameters in SKBR-3 cells which did not show any alteration in elisidepsin sensitivity after four days of hypoxia either (Supplementary Figure S6). These results imply that hypoxia is associated with alterations in the structure of the membrane and the clustering of lipid raft-associated proteins.

## 3. Discussion

The results presented in the paper reveal insight into the mechanism of action of elisidepsin which can be summarized in the following three points: (a) elisidepsin binds to lipid rafts; (b) elisidepsin induces oligomerization of lipid rafts detected in confocal microscopic images and also supported by the fact that elisidepsin-induced membrane permeabilization is characterized by a Hill coefficient of 3–4 while its binding follows a non-cooperative concentration dependence; (c) hypoxia reduces the efficiency of elisidepsin by decreasing its binding to the membrane.

Our results implying the formation of elisidepsin oligomers are in accordance with previous fluorescence resonance energy transfer (FRET) experiments [5]. Here we not only present evidence for the formation of elisidepsin oligomers, but also for elisidepsin-lipid raft interactions and elisidepsin-induced raft clustering. Similar to membrane permeabilization elisidepsin-induced raft associations were induced rapidly, within five min of elisidepsin application followed by the internalization of lipid rafts in 30 min shown in a previous publication [14]. We failed to detect elisidepsin-induced oligomerization of lipid rafts by N&B experiments. We suspect that clusters of GPI-anchored, raft-associated proteins occupy only a small fraction of pixels; therefore, their contribution to the average molecular brightness is negligible. Since they are not immobile for the whole duration of a N&B experiment, they cannot be resolved as pixels with a different molecular brightness either.

Based on the key findings of the paper we propose a model in which elisidepsin binds in a non-cooperative fashion to membrane regions enriched in hydroxylated lipids followed by oligomerization and membrane permeabilization. It was further assumed that elisidepsin oligomers generate the pores responsible for membrane permeabilization and their concentration has to reach a threshold so that necrosis takes place. A quantitative elaboration of the model, provided as Supplementary material, shows that cooperativity in the dose dependence of cell death is the consequence of attributing membrane permeabilization to elisidepsin oligomers [21]. Quantitative predictions of the model which are in accordance with our experimental observations are: (a) elisidepsin binds to the membrane at much lower concentrations than expected based on the killing curves; (b) membrane permeabilization takes place in a narrow concentration range characterized by high cooperativity; (c) the lower the number of elisidepsin binding sites (e.g., in hypoxia or in cells with low FA2H expression), the higher the free concentration of the drug has to be so that the concentration of elisidepsin oligomers in the membrane reaches the critical level; (d) a certain fold-reduction in the number of elisidepsin binding sites results in negligible changes in the IC_50_ if the original number of bindings sites was much higher than the critical level, while the same fold-decrease causes substantial increase in the IC_50_ if the original number of binding sites was close to the critical level (Supplementary Figure S7).

Several lines of evidence support the conclusion that the presence of a membrane environment enriched in hydroxylated lipids is necessary for the binding of elisidepsin: (a) FA2H expression level correlates with elisidepsin sensitivity; (b) hypoxia reduces the efficiency of elisidepsin which is reversed by 2-hydroxy palmitic acid; (c) FA2H expression predicts how much hypoxia increases the IC_50_ of elisidepsin. The last statement can be rationalized by the proposed model since highly expressed FA2H generates more “elisidepsin-friendly” plasma membrane requiring more pronounced hypoxia to reduce the amount of hydroxylated lipids to a level close to the critical concentration. Our results about the role of FA2H in determining elisidepsin sensitivity are in agreement with previous findings [15]. The fact that hydroxylated lipids are primarily present in lipid rafts explains that elisidepsin binds to these membrane microdomains [22]. It has recently been shown that FA2H specifically generates the R-stereoisomer of fatty acids and only the R-enantiomer is able to reverse the effect of FA2H knock-down on membrane viscosity [23]. These findings can be rationalized by the fact that (*S*)-hydroxy fatty acids preferentially form intramolecular hydrogen bonds, while the *R*-stereoisomer is usually involved in intermolecular hydrogen bonds. Therefore, we expect that the (*R*)-2-hydroxy palmitic acid component of the racemic mixture used in our experiments was responsible for restoring the effect of elisidepsin in hypoxic cells.

Hypoxia was found to change the clustering of lipid rafts and the dynamic properties of the cell membrane. Although decreased hydrogen bonding resulting from inhibited hydroxylation is expected to increase membrane fluidity [22], hypoxia decreased the fluidity of the plasma membrane and increased its compactness shown by decreased hydration of Laurdan. These findings are in agreement with previous results showing that hypoxia-induced lipid peroxidation results in decreased membrane fluidity [24,25]. Decreased clustering of GPI-anchored proteins in hypoxic cells may be the consequence of decreased hydrogen bonding or increased viscosity which has been shown to be associated with decreased protein clustering [26].

Lipid hydroxylation is important for the stability of the cell membrane, lipid rafts, myelin sheaths and cornified epithelia [18,22]. The role of FA2H-mediated lipid hydroxylation in maintaining the integrity of certain membranes is supported by observations linking the loss of FA2H expression to late-onset demyelination [27,28]. Cancer cells display characteristic changes in their fatty acid and ganglioside composition [29,30,31]. An increase in the ratio of saturated/non-saturated fatty acids and accumulation of less-complex gangliosides have been observed. In addition, upregulation of FA2H in malignant tumors has been reported which may lead to cancer specific cytotoxic effects of elisidepsin [18,31]. However, tumor hypoxia is known to decrease the rate of hydroxylation due to shortage of oxygen, which acts against the tumor-specificity of elisidepsin by reducing the activity of FA2H [31]. The balance between hypoxia-induced increased FA2H expression and its decreased catalytic activity due to oxygen shortage is unpredictable, but these parameters must be predictive of the sensitivity of tumors to elisidepsin. Although tumor hypoxia is readily detectable *in vivo* [20,32], FA2H expression and the amount of 2-hydroxy fatty acids in the membrane are not amenable to clinical investigations and none of these parameters is routinely measured in clinical practice. But future clinical trials could define the true potential of this drug with a unique mechanism of action in the treatment of human cancer.

## 4. Experimental Section

### 4.1. Cell Culture and Transfection

SKBR-3, HeLa, A431, MCF-7, MDA-MB-453 and CHO cells were obtained from the American Type Culture Collection (ATCC, Manassas, VA, USA) and grown according to their specifications. The immortalized human keratinocyte cell line HaCaT was obtained from the Department of Physiology, University of Debrecen, and cultured in DMEM supplemented with 10% FCS and antibiotics. For generating hypoxic conditions cells plated in a flask or chambered coverglass were kept in a modular hypoxia chamber (Billups-Rothenberg, Del Mar, CA, USA) flushed with a gas mixture containing 1% O_2_, 5% CO_2_ and 94% N_2_ (Linde, Munich, Germany) at a rate of 25 L/min for 4 min. During 4-day hypoxic culturing the cells were not harvested, but they were harvested and split when reaching confluency during 2-week hypoxic culturing. The GFP-GPI plasmid was a kind gift from Jennifer Lippincott-Schwartz (NIH, Bethesda, MD, USA). Cells were transfected with the Amaxa Nucleofector device (Lonza, Basel, Switzerland). The transfection solution and the program were selected according to the “Cell & Transfection Database” of the manufacturer.

### 4.2. Antibodies and Chemicals

The polyclonal antibody against fatty acid 2-hydroxylase (sc161045) and the blocking peptide (sc161045-P) were purchased from Santa Cruz (Santa Cruz, CA, USA). 2-hydroxy and 3-hydroxy palmitic acid were from Sigma-Aldrich (St. Louis, MO, USA). 4′-(trimethylammonio)-diphenylhexatriene (TMA-DPH) and Laurdan (6-dodecanoyl-*N*,*N*-dimethyl-2-naphthylamine) were purchased from Sigma-Aldrich. Elisidepsin was manufactured by PharmaMar (Madrid, Spain) and dissolved in dimethyl sulfoxide at a concentration of 1 mg/mL.

### 4.3. Determination of Elisidepsin Sensitivity

Cells were plated into 96-well plates 24 h before the experiment carried out under normoxic conditions or were kept in hypoxia for 96 h. They were treated with a dilution series of elisidepsin for 30 min in triplicate followed by incubation for 72 h in cell culture medium in a CO_2_ incubator at 37 °C. The viability of cells was determined by measuring the absorbance of WST-1 reagent (Roche Diagnostics GmbH, Mannheim, Germany) with an ELISA reader at 450 nm and 620 nm. The IC_50_ value was determined by fitting the Hill equation to the measurement data using Matlab (Mathworks Inc., Natick, MA, USA).

### 4.4. Flow Cytometric Measurement of Fatty Acid 2-Hydroxylase Expression

Cells were fixed with 3.7% formaldehyde for 30 min on ice followed by washing and labeling with a polyclonal antibody against fatty acid 2-hyroxylase (FA2H) dissolved in PBS containing 0.1% BSA and 0.1% Triton X-100 for 30 min. Unbound antibodies were removed by washing twice in PBS followed by staining with fluorescent secondary antibody. The fluorescence intensity was measured with a FacsArray flow cytometer (Becton Dickinson, Franklin Lakes, NJ, USA). Evaluation was performed with FCS Express (De Novo Software, Los Angeles, CA, USA) and the FA2H levels are reported as the mean intensity of the sample labeled by the primary and the secondary antibodies corrected by subtracting the mean fluorescence intensity of the sample, which was also incubated with the blocking peptide.

### 4.5. Confocal Microscopy

An Olympus FV1000 confocal microscope was used to acquire images using a 60× oil immersion objective (NA = 1.35). GFP-GPI and OregonGreen488 were excited at 488 nm and their emission was measured above 510 nm when the sample was not labeled by any other dye. It was essential to increase the lower cutoff value of the detected emission wavelength range from the default value of 500 nm due to strong light scattering from the glass surface when the membrane adjacent to the coverslip was imaged. AlexaFluor555 was excited at 543 nm and detected above 555 nm. When GFP-GPI and AlexaFluor555 were both present in the sample, the fluorescence of GFP was detected in the spectral region of 510–540 nm. For dual imaging of OregonGreen488-elisidepsin and propidium iodide they were excited at 488 and 543 nm, respectively. The fluorescence of OregonGreen488 was detected in the spectral region of 520 ± 15 nm, whereas propidium iodide was measured at 620 ± 50 nm. Image analysis was carried out with DipImage (Delft University of Technology, Delft, The Netherlands) in a Matlab environment. For quantitative evaluation of membrane-associated fluorescence intensity the cell membrane was identified with the manually-seeded watershed algorithm [33,34] and the mean background corrected fluorescence intensity was calculated. The background was measured in a cell-free area of images. In order to count the number of localized bright spots images were smoothed with a Gauss filter and normalized to their maximum intensity followed by top-hat filtering to remove objects larger than the observed spots. Local maxima were identified by the extended maxima transform followed by filling the holes and shrinking the spots to single points which were enumerated. 

### 4.6. Determination of the Binding of Fluorescent Elisidepsin to the Membrane

For confocal microscopic measurements cells, cultured on chambered coverglass, were labeled with a mixture containing fluorescent and unlabeled elisidepsin at a molar ratio of 1:4 for 20 min in the presence of 10 µg/mL propidium iodide followed by washing to remove unbound elisidepsin. For flow cytometry trypsinized cells were labeled with the same mixture of elisidepsin and measured immediately without washing using a FacsAria instrument (Becton Dickinson). Fluorescent elisidepsin was prepared by labeling the drug with OregonGreen488 or AlexaFluor555 (both from Invitrogen, Carslbad, CA, USA) according to the manufacturer’s specifications. Details of the data analysis are described in Supplementary Materials and Methods (Supplementary Figures S8 and S9).

### 4.7. Number and Brightness (N&B) Analysis of Cells Transfected by GFP-GPI

An Olympus FV1000 confocal microscope running in pseudo photon-counting mode was used to carry out N&B analysis according to Digman *et al*. [35]. Live cells were analyzed at room temperature in Tyrode’s buffer with 10 mM glucose and 0.1% BSA. Image series of 100 optical slices of the cell membrane adjacent to the coverslip were acquired with a pixel size of 82 nm and pixel dwell time of 10 µs. A single image consisted of 256 × 256 pixels and the central part of images was used for analysis to eliminate artifacts arising from scanner speed nonlinearity at the borders. The image stack was analyzed with a custom-written Matlab program incorporating functions of the DipImage toolbox. The images were first registered (*i.e*., corrected for lateral shift) followed by calculating the mean and variance of every pixel. The apparent brightness was calculated according to the following equation:

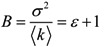
(1)
where *σ*^2^ and ˂*k*˃ are the variance and the mean, respectively, of a given pixel. The molecular brightness (ε) characterizes the clustering state of a fluorescent molecule by giving the number of photons detected from a single diffusing unit during the pixel dwell time. If the image mean decreased by more than 10% due to stage shift or photobleaching or if the pixel variance did not converge to zero with increasing stack size, the stack was discarded.

### 4.8. Measurement of Fluorescence Anisotropy and Generalized Polarization

Trypsinized cells were resuspended in Hank’s buffer at a concentration of 10^7^/mL and labeled with 2 µM TMA-DPH or 2.5 µM Laurdan at room temperature for 20 min. After TMA-DPH labeling cells were diluted in Hank’s buffer without washing to a concentration of 10^6^/mL for fluorescence anisotropy measurements, whereas Laurdan-labeled cells were washed once and resuspended at a concentration of 10^6^/mL in Hank’s buffer. Fluorescence measurements were carried out with a Fluorolog-3 spectrofluorimeter (Horiba Jobin Yvon, Edison, NJ, USA). The temperature of the cuvette holder was adjusted to 37 °C by a circulating water bath. TMA-DPH was excited at 352 nm and its emission was measured at 430 nm. The fluorescence anisotropy (r) of TMA-DPH was measured in the l-format according to the following formula [36,37]:

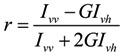
(2)
where *I_vv_* and *I_vh_* are the vertical and horizontal components, respectively, of the fluorescence excited by vertically polarized light, and *G* is a correction factor characterizing the different sensitivity of the detection system for vertically and horizontally polarized light.

Laurdan was excited at 350 nm and its emission was detected in the blue range of its emission spectrum at 435 nm (*I_blue_*) and at the red edge at 500 nm (*I_red_*). Generalized polarization (GP) of Laurdan fluorescence was calculated according to the following formula [38,39,40]: 
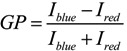
(3)

### 4.9. Determination of 2-Hydroxylated Fatty Acids Using Mass Spectrometry

We used an existing high performance liquid chromatography/mass spectrometry/mass spectrometry (HPLC MS-MS) configuration similar to that already published [41,42]. The separation using HPLC was performed in a manner similar to what has been reported previously [41]. For the detection of 2-hydroxy-palmitate, 3-hydroxy-palmitate and 2-hydroxy-stearate (all chemical reference standards were from Sigma-Aldrich) we established a specific MS-MS method using ESI (−) setting with 271 → 271 *m/z* for 2/3-hydroxy-palmitate with a collision energy of 5 V, a dwell time of 0.1 s and 299 → 299 *m/z* for 2/3-hydroxy-stearate with a collision energy of 5 V, a dwell time of 0.1 s and a cone voltage of 50 V in each case as parameters for multiple reaction monitoring (MRM) measurements.

## 5. Conclusions

In summary, we have shown that the necrotic effect of elisidepsin is highly cooperative which is most likely explained by membrane permeabilization resulting from elisidepsin oligomers. Moreover, we have shown that hypoxia significantly inhibits the anti-tumor effect of elisidepsin in some experimental models, apparently by reducing the level of 2-hydroxy lipids in the membrane of tumor cells. Our results identify tumor hypoxia and the density of 2-hydroxy lipids as factors predicting elisidepsin sensitivity.

## Supplementary Files

Supplementary File 1 Supplementary Materials (PDF, 612 KB)
