# Supplementary material for: Hypoxia Reduces the Efficiency of Elisidepsin by Inhibiting Hydroxylation and Altering the Structure of Lipid Rafts
Source: Mar Drugs. 2013 Dec 2;11(12):4858–75. doi: 10.3390/md11124858 (PMC3877891; doi:10.3390/md11124858)
Supplement: Supplementary File 1 — Supplementary Materials (PDF, 612 KB) [file marinedrugs-11-04858-s001.pdf]

Supplementary Materials

Figure S1. The efficiency of elisidepsin is influenced by hypoxia in certain cell lines. Cells plated in 96-well plates were kept under hypoxic conditions for four days followed by elisidepsin treatment. The normoxic control samples were plated one day before adding elisidepsin. Both normoxic and hypoxic cells were treated with the drug for 30 min and allowed to grow for another three days under normoxic and hypoxic conditions. The symbols show the measured normalized cell number and the continuous lines are fits of the Hill equation to the data points. The IC_{50} values determined by fitting are shown in Table 1.

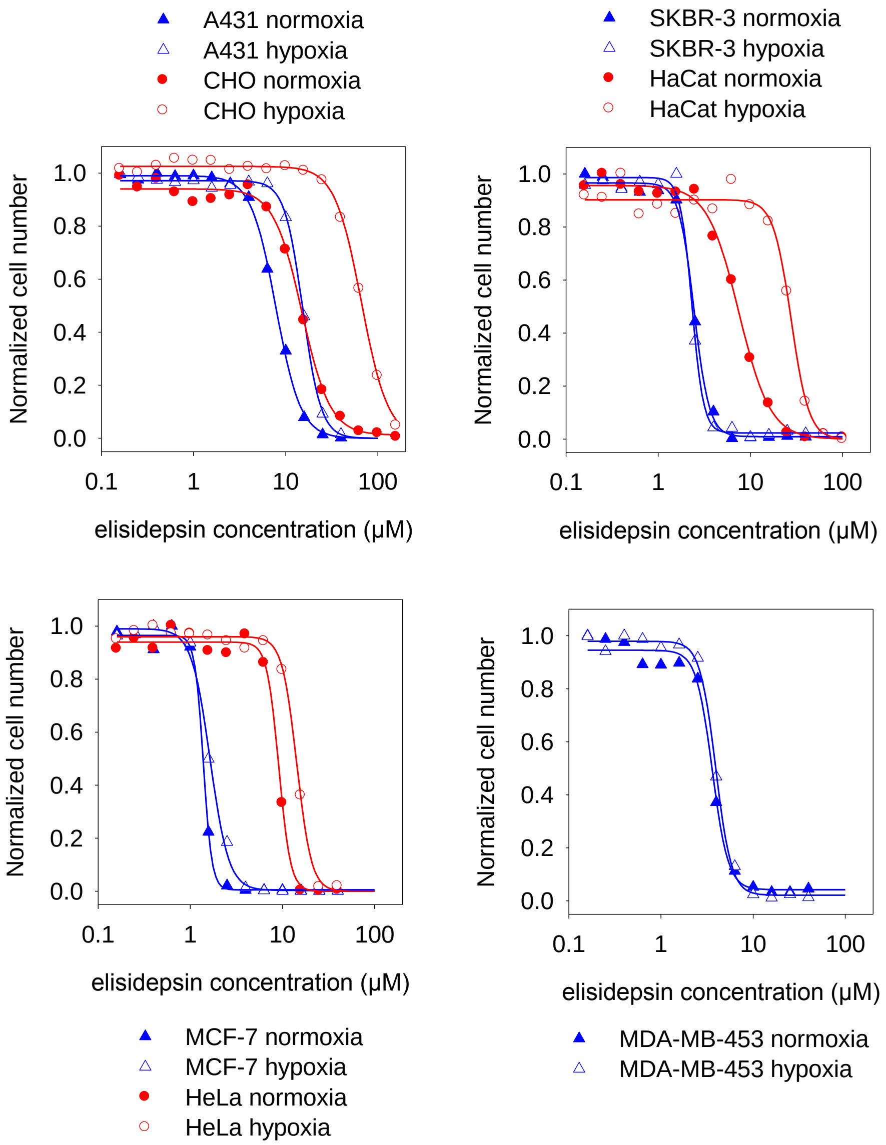

Figure S2. Long-term hypoxia increases the IC_{50} of most sensitive cell lines to elisidepsin. SKBR-3, MDA-MB-453 and MCF-7 cells were cultured under hypoxic conditions for two weeks, while control cells were kept in normoxia. Both of them were treated with different concentrations of elisidepsin for 30 min and allowed to grow for another three days. The continuous lines represent fits of the Hill equation to the measured data.

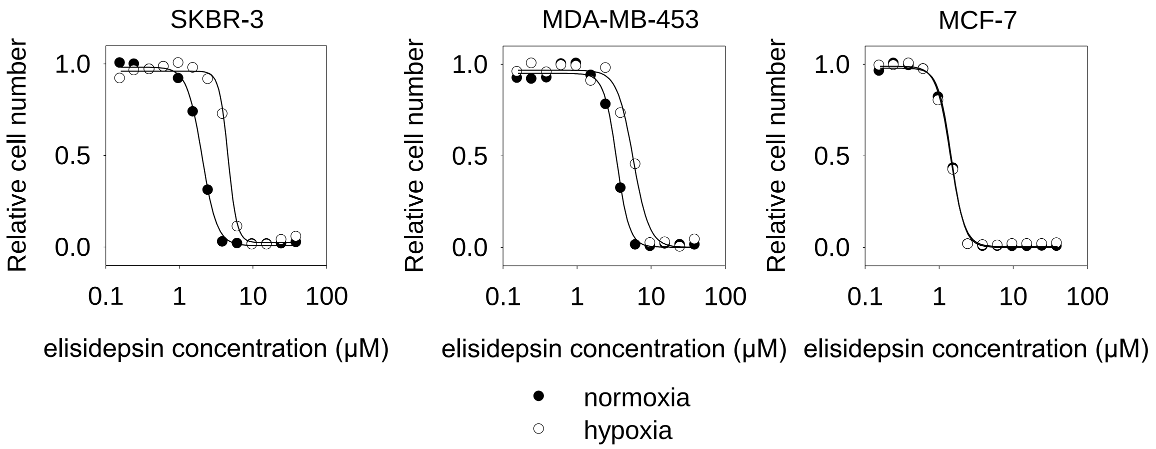

Figure S3. Determination of standard compounds and normoxic as well as hypoxic cells using HPLC-MS-MS. Cells kept under normoxic conditions or cultured in hypoxia for four days were pelleted and equal amounts were measured using mass spectrometry. The data of normoxic and hypoxic cells are displayed on the same vertical scale; therefore, the quantities of the compounds in the cells under different conditions are comparable. Hypoxic conditions resulted in decreased elisidepsin sensitivity paralleled by decreased 2-hydroxy palmitic acid (2-OH-C16) and 2-hydroxy stearic acid (2-OH-C18) levels without significant changes in the amount of 3-hydroxy fatty acids.

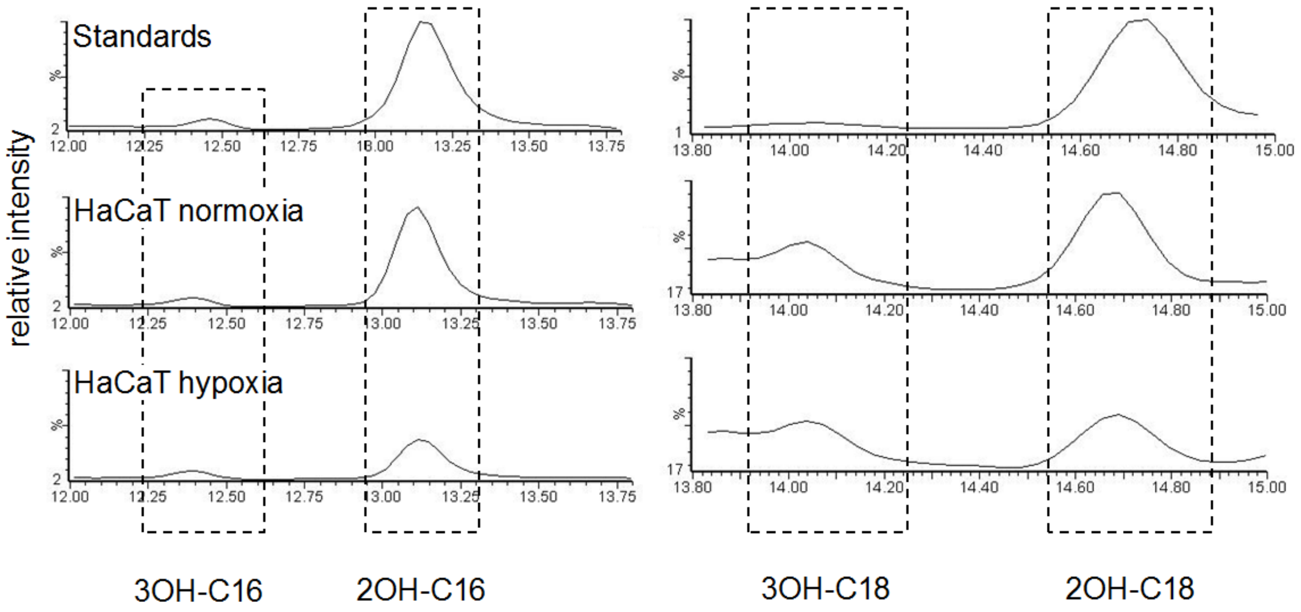

Figure S4. 2-hydroxy palmitic acid reverses the effect of hypoxia on the elisidepsin sensitivity of cell lines. Cells plated in 96-well plates were kept under hypoxic conditions for four days. On the third day they were treated with 100 µM 2-hydroxy palmitic acid (2-OH-PA) or its solvent, methanol (MeOH). The normoxic control samples were plated one day before adding elisidepsin. Both normoxic and hypoxic cells were treated with the drug for 30 min and allowed to grow for another three days under normoxic and hypoxic conditions. The symbols show the measured normalized cell number and the continuous lines are fits of the Hill equation to the data points. The calculated IC_{50} values are displayed in Table 2.

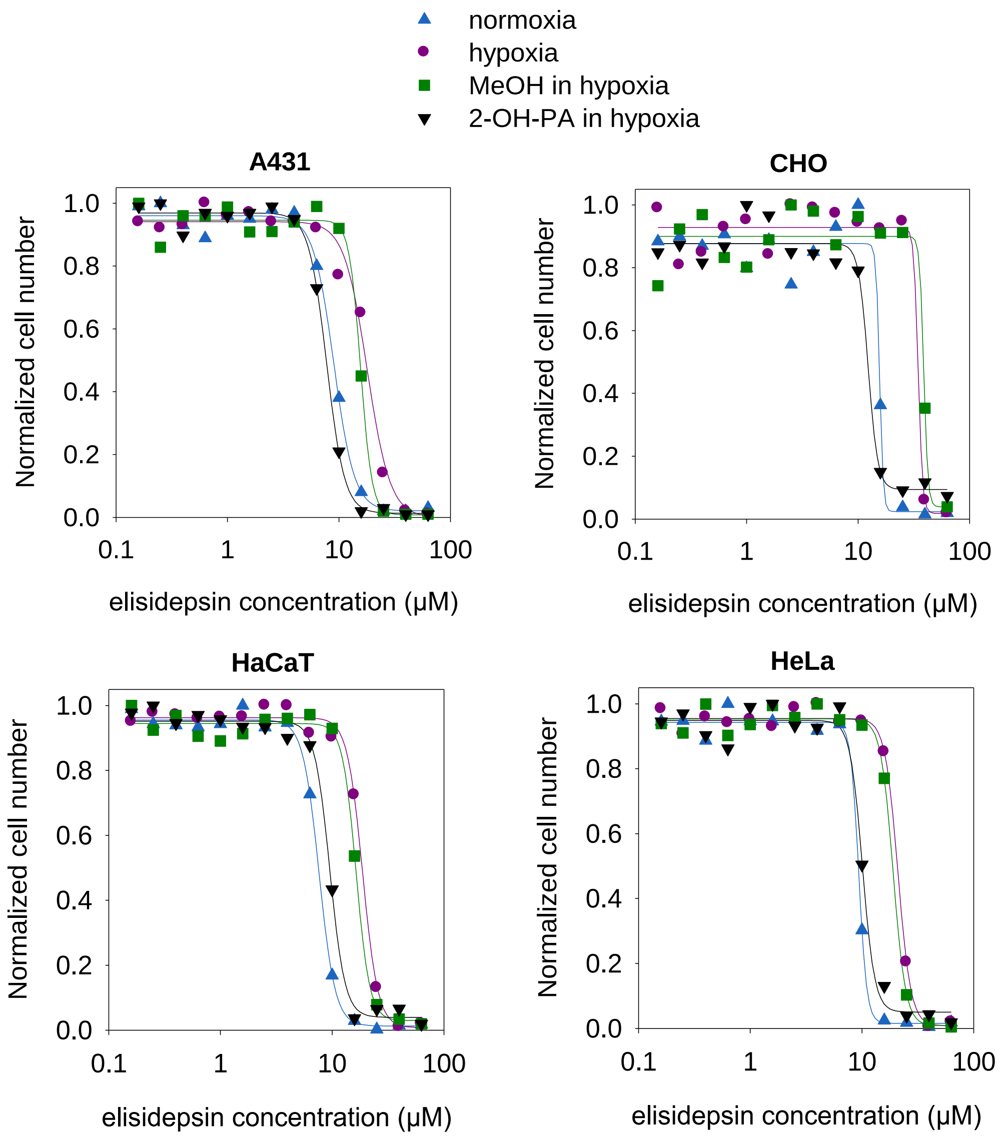

Figure S5. Differences in the concentration dependence of the binding and killing effect of elisidepsin. A431 cells were treated with elisidepsin ((**A**) – 0.36 μM; (**B**) – 1 μM; (**C**) – 2.5 μM; (**D**) – 5 μM; (**E**) –10 μM; (**F**) – 44 μM) containing a 1:4 mixture of OregonGreen488 elisidepsin and unlabeled drug for 20 min in the presence of 10 μg/mL propidium iodide followed by washing and confocal microscopy. The elisidepsin and propidium iodide fluorescence intensities are displayed in the green and red channels, respectively. Quantitative analysis of the images is shown in Figure 4.

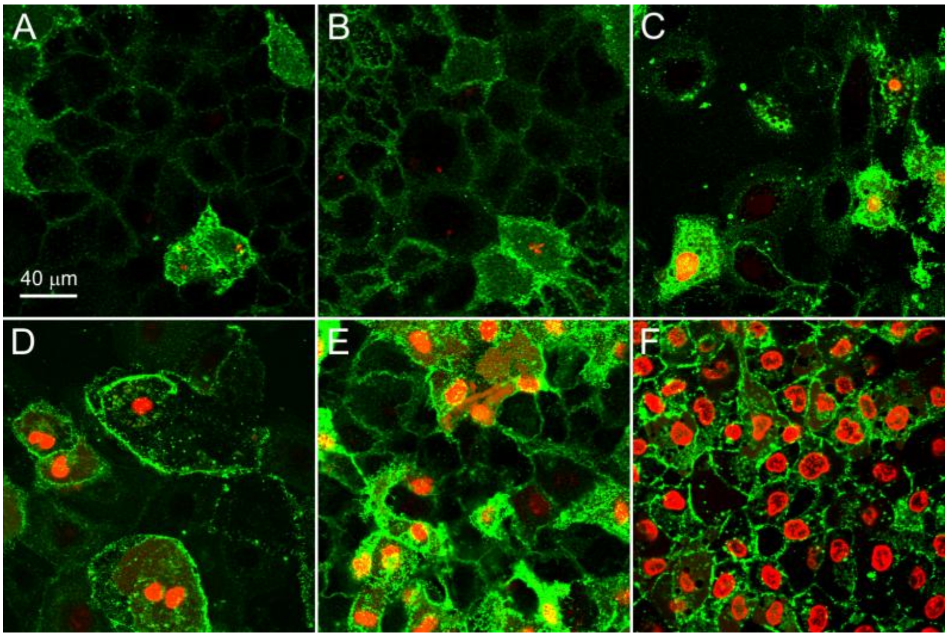

Figure S6. Hypoxia results in decreased fluidity and increased compactness of the membrane. A431 and SKBR-3 cells were cultured under hypoxic conditions for four days followed by labeling with TMA-DPH for fluorescence anisotropy measurements (**A**) or with Laurdan for measurement of generalized polarization (**B**). The mean (±standard error of the mean) of five independent measurements is shown. Asterisks indicate significant difference between normoxic and hypoxic samples ($p < 0.05$).

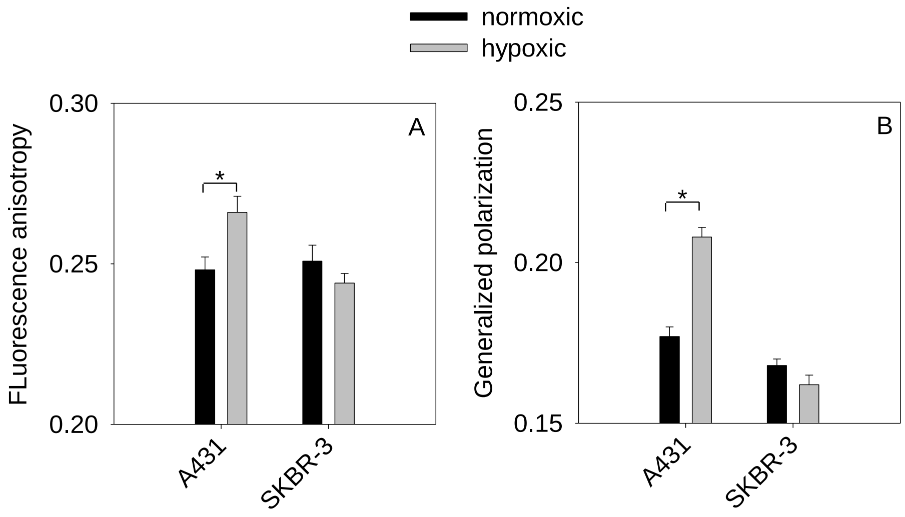

S1. Theory

Quantitative Model Predicting the Fraction of cells Killed by Elisidepsin

We created a model in which the following assumptions were made (model 1): (a) Elisidepsin binds in a non-cooperative fashion to the membrane; (b) Membrane-bound monomeric elisidepsin undergoes stepwise oligomerization; (c) n-mers generated by the stepwise oligomerization form pores in the membrane; (d) cells in which the number of pores exceeds a certain critical level are killed.

Non-cooperative binding of elisidepsin to the membrane is described by the following equation:

$$E_{free}L_h = E_1 K_{db} \quad \text{(S1)}$$

where E_1 is the membrane-bound concentration of monomeric elisidepsin and E_{free} is the free concentration of the drug in the solution. The binding is characterized by the dissociation constant K_{db}. L_{h} is the concentration of hydroxylated lipids, the assumed binding site of elisidepsin, in the membrane. Membrane-bound elisidepsin was assumed to undergo stepwise oligomerization according to the following equations:

$$\begin{aligned} E_1 E_1 &= K_d E_2 \\ E_2 E_1 &= K_d E_3 \\ &\ldots \\ E_{n-1} E_1 &= K_d E_n \end{aligned} \quad \text{(S2)}$$

where E_2, E_3, … E_{n} are the concentrations of dimers, trimers and n-mers of elisidepsin, respectively. Consequently, the concentration of E_{n} can be expressed according to the following equation:

$$E_n = \frac{E_1^{\,n}}{K_d^{\,n-1}} \quad \text{(S3)}$$

Since a dimer, trimer, …, n-mer contains two, three, …, *n* elisidepsin monomers and hydroxylated lipid binding sites, the number of monomers and binding sites in an n-mer is given by the following equation:

$$N_n = \frac{nE_1^{\,n}}{K_d^{\,n-1}} \quad \text{(S4)}$$

The concentrations N_1, N_2 to N_{n} in Equation S10 constitute a power series in which the sum of the first *n* terms (N_1 to N_{n}) can be expressed by the following expression:

$$\sum_{k=1}^{n} N_k = E_1 \frac{1-(n+1)\left(\frac{E_1}{K_d}\right)^n + n\left(\frac{E_1}{K_d}\right)^{n+1}}{\left(1-\frac{E_1}{K_d}\right)^2} \quad \text{(S5)}$$

It follows that the total concentration of hydroxylated lipid binding sites in the system is the sum of the concentration of unsaturated binding sites (L_h) and the concentrations of monomers and n-mers in the membrane (Equation S11):

$$L_{h,tot} = L_h + \sum_{k=1}^{n} N_k \tag{S6}$$

Equations S7, S9 and S12 were solved for E_n and E_{free} as a function of E_1:

$$E_n = E_1^{\,n} K_d^{\,1-n}$$

$$E_{free} = -\frac{E_1 \left(E_1 - K_d\right)^2 K_{db}}{E_1 K_d^{\,2} - E_1^{\,2} L_{h,tot} + 2E_1 K_d L_{h,tot} - K_d^{\,2} L_{h,tot} + E_1 \left(\frac{E1}{K_d}\right)^n K_d \left(E_1 n - K_d \left(1+n\right)\right)} \tag{S7}$$

A step function was defined providing the fraction of killed cells as a function of E_n:

$$F_{killed} = \begin{cases} 0, \text{ if } E_n < c_{crit} \\ 1, \text{ if } E_n \geq c_{crit} \end{cases} \tag{S8}$$

where c_{crit} is the critical concentration of pores above which a cell is killed by elisidepsin.

Parametric plots of F_{killed} *vs*. E_{free} were created showing that if $L_{h,tot} >> c_{crit}$, a moderate (25%) decrease in the number of binding sites results in negligible changes in IC_{50}, whereas if the number of binding sites ($L_{h,tot}$) approaches the critical number of pores (c_{crit}), the same fold-decrease in the number of binding sites leads to an extreme increase in IC_{50} (Figure S7A).

In order to generate curves without a discontinuity two further assumptions were made (model 2): (a) the number of binding sites ($L_{h,tot}$) was assumed to follow a normal distribution; (b) in order to obviate the need to solve a general nth order polynomial equation, it was assumed that elisidepsin undergoes trimerization in the membrane. The same equation set was solved, but this time for E_n as a function of E_{free}. The dependence of IC_{50} on the number of elisidepsin binding sites showed a behavior similar to the one discussed above (Figure S7B). Although other sources of biological variability, e.g., cell size, metabolism of hydroxylated fatty acids, vesicular transport, signal transduction pathways promoting cell survival, obviously affect the IC_{50} of elisidepsin, we can conclude that the proposed model recapitulates the observations that (a) there is a steep increase in the fraction of killed cells at around the half-maximal inhibitory concentration, *i.e.*, large Hill coefficient; (b) hypoxia-induced decrease in the number of binding sites results in negligible changes in the IC_{50} if the number of binding sites (hydroxylated lipids) is much larger than the critical concentration required for cell death.

Figure S7. Hypoxia-induced increase in the IC_{50} value of elisidepsin depends on the relationship between the number of hydroxylated lipids to the critical concentration of pores in the membrane. (**A**) Parametric plots were made according to model 1 using the following parameters: K_d = 300, K_{db} = 10, c_{crit} = 100, n = 3 and $L_{h,tot}$ as displayed in the figure; (**B**) Plots of the fraction of killed cells as a function of free elisidepsin concentration were generated according to model 2 using the previous parameters and assuming a Gaussian distribution of binding sites characterized by a standard deviation equal to one-tenth of the mean.

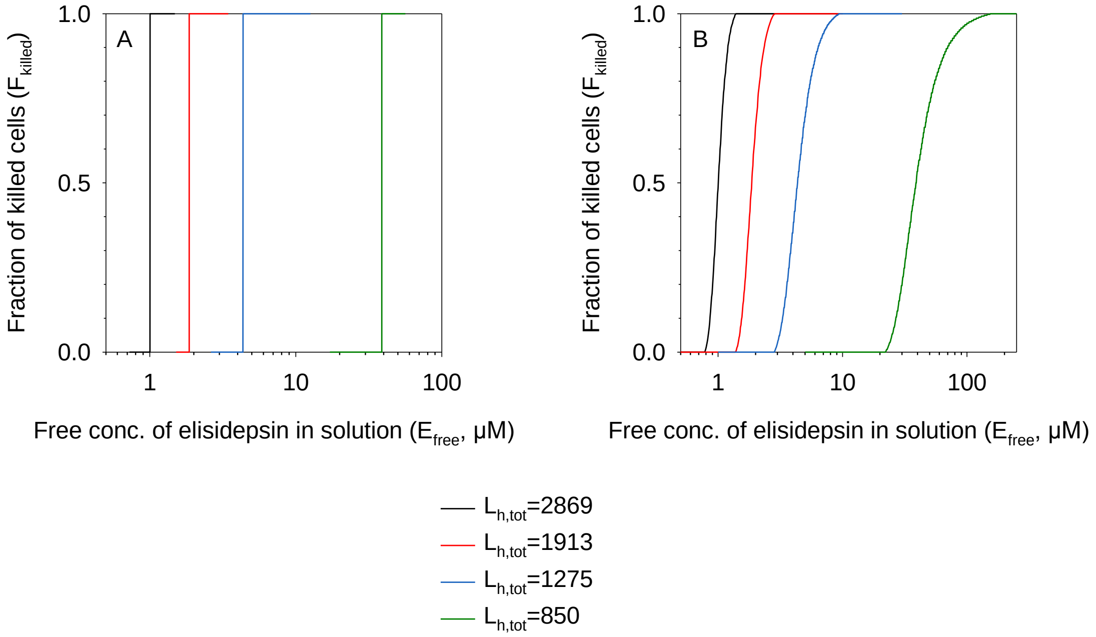

All calculations were carried out in Mathematica.

S2. Supplementary Materials and Methods

Quantitative analysis of elisidepsin fluorescence intensity and the fraction of propidium iodide-positive cells in confocal microscopy: Cells grown on coverslips were incubated with 10 μg/mL propidium iodide and elisidepsin for 20 min. Elisidepsin was a 1:4 molar mixture of OregonGreen488-conjugated and unlabeled drug. Images were taken in the propidium iodide and OregonGreen488 channels by confocal microscopy. Image processing was carried out in DipImage (Delft University of Technology, Delft, The Netherlands) under Matlab (Mathworks Inc., Natick, MA, USA). The propidium iodide image was thresholded. Four different histogram-based segmentations were tested (manual thresholding, the intermeans algorithm, the maximum entropy algorithm, Otsu's method) with all of them yielding visually indiscriminatable results [1]. The resultant image was processed by the binary closing algorithm to remove holes followed by binary opening to remove small objects. Finally, the foreground patches were shrunk to single pixels. Their sum yielded the number of detectable nuclei in the image. Membranes in the OregonGreen488 image were identified by the manually-seeded watershed segmentation algorithm [2]. The total number of cells in the image was determined by counting the number of closed circumferences in the image after manual removal of non-cell objects. The relative fraction of propidium iodide positive cells was calculated by dividing the number of nuclei in the propidium

iodide image by the number of cells identified in the OregonGreen488 image. Steps of the image processing are shown in Figure S8.

Figure S8. Segmentation of images of cells labeled with propidium iodide and fluorescent elisidepsin. A431 cells were incubated in the presence of 10 μg/mL propidium iodide and 10 μM elisidepsin in which OregonGreen488-elisidepsin and unlabeled elisidepsin were mixed in a 1:4 molar ratio. Images were recorded after 20 min of incubation. The propidium iodide image (**A**) was thresholded (**B**) followed by binary closing (**C**) and opening (**D**). Finally, the foreground patches were shrunk to single pixels whose size was increased for visualization. These points are shown overlaid on the original propidium iodide image in (**E**). Images recorded in the OregonGreen488 channel (**F**) were segmented using the manually seeded watershed transformation yielding the membrane mask shown in red overlaid on the original image in (**G**). The segmented membranes (black lines), cells (colored objects) and nuclei (black circles) are displayed in part (**H**).

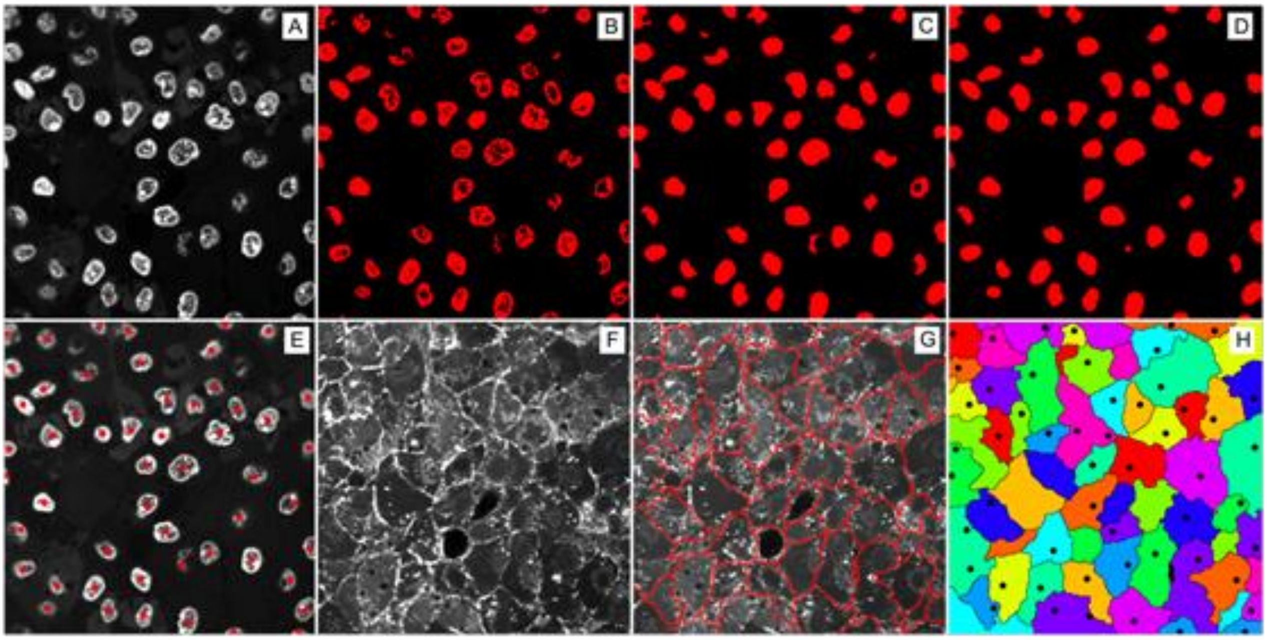

Flow cytometric estimation of binding of fluorescent elisidepsin to the membrane: Trypsinized cells were incubated with a 1:4 molar mixture of OregonGreen488-conjugated and unlabeled elisidepsin (total drug concentration was 0.5 μM) and measured immediately without washing using flow cytometry. Intensities were recorded in a time-correlated manner. The aim of the experiment was to measure the fluorescence intensity of membrane-bound elisidepsin. The built-in background subtraction algorithm of the flow cytometer removed the intensity of unbound elisidepsin, but the fluorescence of intracellular and membrane-bound molecules could not be differentiated from each other. However, elisidepsin first binds to the membrane followed by internalization; therefore the initial part of the binding curve represents membrane-bound fluorescence. Since it is difficult to determine the time at which elisidepsin starts to get internalized, the slope of the initial part of the binding curve was determined, which represents the binding of the drug to the membrane according to the following derivation (Figure S9).

Figure S9. Determination of the slope of the fluorescence intensity trend line in a time-correlated flow cytometric measurement. The graph shows a representative measurement of time-dependent increase of fluorescence intensity in a binding experiment. In addition to the fluorescence intensity the time of measurement was also recorded and the time-dependent increase in the fluorescence intensity of individual cells was plotted (black dots). The red trend line shows the average fluorescence intensity calculated every 0.25 s and the blue line is result of linear regression performed on cells recorded in the first 5 s.

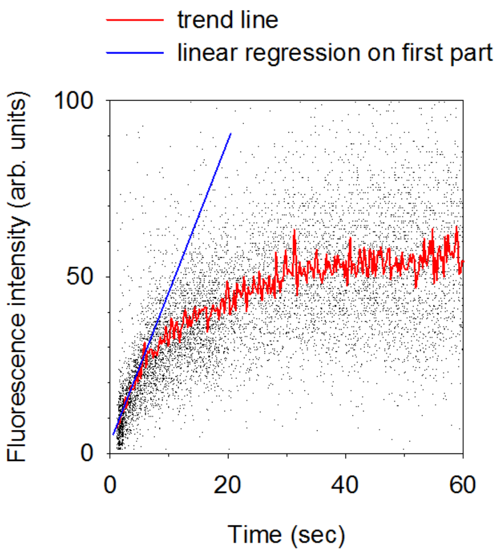

The binding of elisidepsin to the membrane is described by the following set of differential equations:

$$\frac{dER(t)}{dt} = E_f(t)R_f(t)k_{on} - ER(t)k_{off} \quad \text{(S9)}$$

$$\frac{dR_f(t)}{dt} = -\frac{dER(t)}{dt} \quad \text{(S10)}$$

$$E_f(t) = E_{tot} \quad \text{(S11)}$$

$$ER(0) = 0 \quad \text{(S12)}$$

$$R_f(0) = R_{tot} \quad \text{(S13)}$$

where *ER* is the complex of elisidepsin with its receptor, E_f and R_f are the free drug concentration and the free receptor concentration, respectively, and R_{tot} is the total receptor concentration. k_{on} and k_{off} are the on-rate and off-rate, respectively. Ligand depletion was assumed to be negligible therefore the free drug concentration (E_f) is equal to the total drug concentration (E_{tot}). Although the solution of the equation set for *ER*(t) is an exponential function of time, its initial part (where elisidepsin is assumed to bind exclusively to the membrane) can be approximated with a straight line:

$$ER(t) = \frac{\left(1 - e^{-(k_{off} + k_{on}E_{tot})t}\right)k_{on}E_{tot}R_{tot}}{k_{off} + k_{on}E_{tot}} \approx k_{on}E_{tot}R_{tot}\,\mathrm{t} \text{ if } t \to 0 \quad \text{(S14)}$$

The slope of the linear part is proportional to the product $k_{on}{\cdot}R_{tot}$ which represents the avidity of binding of elisidepsin to the membrane.

Derivation of the equation was carried out with Mathematica (Wolfram Inc., Champaign, IL, USA), whereas determination of the slope was done by exporting list-mode data from FCS Express (Denovo Software, Los Angeles, CA, USA) to Microsoft Excel and performing linear regression on the initial part of the curve.

References

1. Gonzalez, R.C.; Woods, R.E.; Eddins, S.L. Thresholding. In *Digital Image Processing Using Matlab*; Gonzalez, R.C., Woods, R.E., Eddins, S.L., Eds.; Pearson Prentice Hall: Upper Saddle River, NJ, USA, 2004; pp. 404–407.
2. Gonzalez, R.C.; Woods, R.E.; Eddins, S.L. Segmentation Using the Watershed Algorithm. In *Digital Image Processing Using Matlab*; Gonzalez, R.C., Woods, R.E., Eddins, S.L., Eds.; Pearson Prentice Hall: Upper Saddle River, NJ, USA, 2004; pp. 417–425.

© 2013 by the authors; licensee MDPI, Basel, Switzerland. This article is an open access article distributed under the terms and conditions of the Creative Commons Attribution license (http://creativecommons.org/licenses/by/3.0/).
